# Vitamin D and HIV Progression among Tanzanian Adults Initiating Antiretroviral Therapy

**DOI:** 10.1371/journal.pone.0040036

**Published:** 2012-06-29

**Authors:** Christopher R. Sudfeld, Molin Wang, Said Aboud, Edward L. Giovannucci, Ferdinand M. Mugusi, Wafaie W. Fawzi

**Affiliations:** 1 Department of Epidemiology, Harvard School of Public Health, Boston, Massachusetts, United States of America; 2 Department of Biostatistics, Harvard School of Public Health, Boston, Massachusetts, United States of America; 3 Department of Nutrition, Harvard School of Public Health, Boston, Massachusetts, United States of America; 4 Department of Global Health and Population, Harvard School of Public Health, Boston, Massachusetts, United States of America; 5 Department of Microbiology and Immunology, Muhimbili University of Health and Allied Sciences, Dar es Salaam, Tanzania; 6 Department of Internal Medicine, Muhimbili University of Health and Allied Sciences, Dar es Salaam, Tanzania; 7 Channing Laboratory, Department of Medicine, Brigham and Women’s Hospital, Boston, Massachusetts, United States of America; University of Toronto, Canada

## Abstract

**Background:**

There is growing evidence of an association between low vitamin D and HIV disease progression; however, no prospective studies have been conducted among adults receiving antiretroviral therapy (ART) in sub-Saharan Africa.

**Methods:**

Serum 25-hydroxyvitamin D (25(OH)D) levels were assessed at ART initiation for a randomly selected cohort of HIV-infected adults enrolled in a trial of multivitamins (not including vitamin D) in Tanzania during 2006–2010. Participants were prospectively followed at monthly clinic visits for a median of 20.6 months. CD4 T-cell measurements were obtained every 4 months. Proportional hazard models were utilized for mortality analyses while generalized estimating equations were used for CD4 T-cell counts.

**Results:**

Serum 25(OH)D was measured in 1103 adults 9.2% were classified as vitamin D deficient (<20 ng/ml), 43.6% insufficient (20–30 ng/mL), and 47.2% as sufficient (>30 ng/mL). After multivariate adjustment, vitamin D deficiency was significantly associated with increased mortality as compared to vitamin D sufficiency (HR: 2.00; 95% CI: 1.19–3.37; p = 0.009), whereas no significant association was found for vitamin D insufficiency (HR: 1.24; 95% CI: 0.87–1.78; p = 0.24). No effect modification by ART regimen or change in the associations over time was detected. Vitamin D status was not associated with change in CD4 T-cell count after ART initiation.

**Conclusions:**

Deficient vitamin D levels may lead to increased mortality in individuals receiving ART and this relationship does not appear to be due to impaired CD4 T-cell reconstitution. Randomized controlled trials are needed to determine the safety and efficacy of vitamin D supplementation for individuals receiving ART.

## Introduction

In 2002 only 2% of HIV-infected individuals eligible for antiretroviral therapy (ART) in sub-Saharan were receiving treatment, while by the end of 2010 this proportion increased to 49% with over 5 million people receiving ART [1]. Despite successes in expanding treatment coverage, individuals initiating ART in sub-Saharan Africa experience high mortality rates and interventions are needed to prolong and improve quality of life [2], [3].

Vitamin D is a potent immunomodulator with effects on both adaptive and innate immune responses [4], [5]. Vitamin D may play a particularly strong role in control of intracellular pathogens by enhancing cell-mediated immunity, production of antimicrobial peptides, and phagocytic activity of macrophages [6]–[9]. As a result, HIV-infected individuals with adequate levels of vitamin D may better control HIV or opportunistic infections compared to deficient individuals [10]. Further, vitamin D has effects on multiple organ systems and HIV-infected individuals with low levels of vitamin D could experience increased complications of ART including: cardiovascular disease, insulin resistance, and renal impairment [11]–[13].

Three previous cohorts have investigated the association of vitamin D status and HIV mortality; however, each of these studies has methodological or generalizability limitations. The first small study conducted in Norway before introduction of ART found that HIV-infected individuals with low 1, 25(OH)_2_D (active form of vitamin D) had significantly decreased survival time compared to individuals with normal levels [14]. However, serum 1,25(OH)_2_D is considered a sub-optimal measure of vitamin D status and serum 25-hydroxyvitamin D (25(OH)D) is the only vitamin D metabolite that should be employed to assess vitamin D status [15]. The second study, consisting of HIV-infected pregnant women in Tanzania not receiving ART, found women in the highest quintile of 25(OH)D, had 42% lower risk of all-cause mortality as compared to those in the lowest quintile [16]. Low vitamin D was also associated with wasting, anemia, and incidence of acute upper respiratory infections in these women [17], [18]. Nevertheless, these results may not be generalizable to men and non-pregnant women receiving ART since pregnancy and antiretroviral drugs can interact with vitamin D metabolism and the role of vitamin D may be altered during immune reconstitution [19], [20]. The third cohort study consisted of adults in 31 European countries, Israel, and Argentina who were mostly receiving ART and similarly found adults in the highest tertile of 25(OH)D had a 0.56 times the rate of mortality compared to individuals in the lowest tertile [21]. However, these finding may not be generalizable to individuals receiving ART in resource-limited countries. Individuals receiving ART in developing countries initiate at significantly lower CD4 T-cell counts and have higher risk of comorbid infections, which may modify the impact of vitamin D on mortality [22].

To date no longitudinal studies of vitamin D and HIV disease progression have been conducted among adults receiving ART in sub-Saharan Africa or for men and non-pregnant women in a resource-limited setting. Examining the relationship between vitamin D and HIV disease progression in this population is essential given over one million adults initiated ART in sub-Saharan Africa in 2010 and coverage is rapidly expanding [1]. Furthermore, no studies have investigated the impact of vitamin D on change in CD4 T-cell count in HIV-infected individuals receiving ART in a resource-limited setting where individuals start ART at low CD4 T-cell counts, which will give valuable insight to the mechanism of vitamin D on mortality. Here we present a prospective cohort study of Tanzanian adults initiating ART to address these knowledge gaps.

## Materials and Methods

### Study Population

This prospective cohort study consisted of randomly selected sample of HIV-infected men and women initiating ART enrolled in a double-blind, randomized controlled trial assessing the effect of daily oral supplements of vitamins B-complex, C, and E at high versus standard levels of the recommended dietary allowance (RDA) on HIV disease progression conducted in Dar es Salaam, Tanzania during 2006–2010. Participants were recruited from 7 HIV care and treatment centres in Dar es Salaam with the support of the National AIDS Control Program and the President’s Emergency Plan for AIDS Relief program and in collaboration with the Harvard School of Public Health, Muhimbili University of Health and Allied Sciences, and the City of Dar es Salaam Regional Office of Health.

Individuals were eligible for the study if they were aged ≥18 years, HIV-infected, initiating ART at enrollment, and intended to stay in the city of Dar es Salaam for at least 2 years. Women who were pregnant or lactating were excluded from the study. At the time of the study, patients with World Health Organization (WHO) HIV disease stage IV, CD4 T-cell count <200 cells/ µL, or with WHO HIV stage III disease and CD4 T-cell count <350 cells/ µL were initiated on HAART [23]. First-line drug combinations included stavudine (d4T), lamivudine (3TC), nevirapine (NVP), zidovudine (AZT), and efavirenz (EFV). AZT was substituted for d4T for individuals with peripheral neuropathy or who could not tolerate d4T. EFV was substituted for NVP in patients who could not tolerate NVP. Therefore four different ART regimens were used in trial: (i) d4T - 3TC - NVP, (ii) d4T - 3TC - EFV, (iii) AZT - 3TC - NVP, (iv) AZT - 3TC - EFV. Co-trimoxazole prophylaxis was provided when CD4 T-cell counts were <200 cells/ µL, and treatment for all opportunistic infection was provided according to the national and WHO guidelines.

### Baseline Covariate Assessment

At enrollment, a full clinical examination was conducted and a structured interview was completed to collect information on demographic characteristics. Season of the baseline visit categorized by long rain (December-March), harvest (April-May), post-harvest (June-August), and short rain (September-November). Study physicians performed a complete medical examination and assessed HIV disease stage in accordance with the WHO guidelines, and collected blood specimens. Absolute CD4 T-cell count (FACSCalibur flow cytometer, Becton Dickinson, San Jose, CA) and complete blood count (AcT5 Diff AL analyzer, Beckman Coulter, Miami, FL) were also determined. Height/weight measurements were obtained by trained research assistants using standardized procedures and calibrated instruments.

### Vitamin D Assessment

Blood samples were obtained from study participants at baseline and plasma was stored at or below −70°C. 25(OH)D was quantified by high performance liquid chromatography tandem mass spectrometry (HPLC-MS/MS) using an API-5000 (AB Sciex, Foster City, CA) at Children’s Hospital Boston [24]. Serum samples were first extracted and ‘cleaned up’ using the Aria-TLX-2 (Thermo Fisher Scientific, Waltham, MA) after which 50 µL was mixed with acetonitrile containing internal standard of 25-hydroxyviatmin d6D3. Samples were then centrifuged and 50 µL of the supernatant was injected into the Aria-TLX-2, passed through a Cyclone-P column (Thermo Fisher Scientific), and then eluted through a Kinetex C column (Phenomenex, Torrance, CA). The eluate was injected into the API-5000 for atmospheric pressure chemical ionization and passed through the triple quadrupole mass spectrometer for detection and quantified measurements. The assay is linear up to 100 ng/mL, and sensitive to 1 ng/mL. Day-to-day precision (%CV) at various levels of 25(OH)D ranged from 5.6% to 8.5%.

### Outcome Assessment

Participants were followed at monthly clinic visits where physicians performed a clinical evaluation and a nurse assessed self-reported symptoms and HIV-related complications since the last visit. Absolute CD4 T-cell count was determined every 4 months. Participants who missed a clinic visit were followed at home where relatives or neighbors were asked about vital status of the participant.

### Statistical Methods

We examined risk factors for low vitamin D status (25(OH)D <30 ng/mL) at ART initiation using binomial regression models in order to obtain risk ratio estimates [25], [26]. In a few instances, the models did not converge and log-Poisson models, which provide consistent but not fully efficient estimates of the relative risk and its confidence intervals were used [27]. Variables included in this analysis included: sex, age, season, body mass index (BMI), WHO HIV disease stage, baseline CD4 T-cell count, and hemoglobin levels.

The relationship of vitamin D levels at ART initiation and mortality was investigated using proportional hazard models [28]. There is not absolute consensus on the ideal 25(OH)D level and as a result we analyzed 25(OH)D levels categorically with clinical cut-offs as well as continuously [29], [30]. We defined vitamin D deficiency as 25(OH)D concentrations <20 ng/mL, insufficiency 20–30 ng/mL, and sufficiency as >30 ng/mL [31]–[33]. The possible non-linear relation between serum 25(OH)D and mortality was also examined non-parametrically with restricted cubic splines [34], [35]. Tests for non-linearity used the likelihood ratio test, comparing the model with only the linear term to the model with the linear and the cubic spline terms. Individuals without events were censored at the date of last follow-up visit. Confounders considered for multivariate mortality models included variables with univariate p-values <0.20 in the risk factor analysis or variables noted to be significant confounders in previous studies were selected for inclusion.

Associations of vitamin D status and CD4 T-cell counts were analyzed with generalized estimating equations. Change in CD4 T-cell count between consecutive visits was treated as a longitudinal continuous outcome and vitamin D, time since ART initiation, along with other baseline covariates as explanatory variables. We used an m-dependent working correlation matrix (m = 1) assuming the correlation coefficient of adjacent observations are non-zero and equal, and robust estimators of the variances were utilized to construct confidence intervals. The robust estimators are consistent estimators of the variances even if the working correlation matrix is misspecified. The potential non-linear relationship of change in T-cell counts between consecutive visits over time was examined non-parametrically with restricted cubic splines for time since ART initiation [34], [35]. If a non-linear relationship was found, we added the selected cubic spline terms to the above-specified model as covariates. To assess whether vitamin D status was associated with change in CD4 T-cell between consecutive visits over time, we included the interactions of vitamin D status with time since ART initiation and its splines. The robust score test was used to determine whether vitamin D deficient and insufficient individuals differed in CD4 T-cell trajectory as compared to sufficient individuals. Confounders were also selected from the risk factor analysis; however, baseline CD4 T-cell count was not adjusted for as it will result in biased estimates [12].

Effect modification by all covariates, randomized multivitamin regimen, and ART regimen were considered for all analyses. Interaction by ART regimen was examined for each of the four regimen combinations and also by antiretroviral drug (i.e. efavirenz-containing regimen). To determine whether effect modification was statistically significant, we used the likelihood ratio test in proportional hazard models and the robust score test in GEE analyses. Missing data for covariates was retained in the analysis using the missing indicator method for variables missing greater than 1% of the observations [36]. All p-values were 2-sided and p<0.05 considered statistically significant. Statistical analyses were performed using the SAS v 9.2 (SAS Institute Inc., Cary, NC, USA).

### Ethics Statement

Written informed consent was obtained from all participants included in the parent trial. The trial protocol was approved by the institutional review boards of the Harvard School of Public Health, Muhimbili University of Health and Allied Sciences, Tanzania Food and Drug Authority and National Institute of Medical Research.

## Results

A total of 1103 individuals were randomly selected for 25(OH)D testing from 3,418 individuals enrolled in the parent trial. There were no significant differences between the randomly selected cohort and the trial population. 101 (9.2%) individuals were classified as vitamin D deficient (<20 25(OH)D ng/mL), 481 (43.6%) as insufficient (20–30 ng/mL), and 521 (47.2%) as vitamin D sufficient (>30 ng/mL). Baseline characteristics of the cohort at ART initiation visit are presented in Table 1. A multivariate risk factor analysis determined low vitamin D (25(OH)D <30 ng/mL) was independently associated with younger age (p<0.001), increased CD4 T-cell count (p<0.001), and season of the ART initiation visit (Table 2).

**Table 1:** Characteristics of Vitamin D cohort at baseline ART Initiation visit by vitamin D status.

	Vitamin D Deficient<20 ng/mL (n = 101)	Vitamin D Insufficient20–30 ng/mL (n = 481)	Vitamin D Sufficient>30 ng/mL (n = 521)
	Mean ± SD or Frequency (%)	Mean ± SD or Frequency (%)	Mean ± SD or Frequency (%)
Female	372 (71.3)	336 (69.9)	351 (67.4)
Age (years)			
Under 30	18 (17.8)	85 (17.7)	58 (11.1)
30–40	56 (55.4)	241 (50.1)	249 (47.8)
40–50	17 (16.8)	110 (22.9)	167 (32.1)
Over 50	10 (9.9)	45 (9.4)	47 (9.0)
Season			
Long Rain (Dec-Mar)	9 (8.9)	58 (12.1)	83 (15.9)
Harvest (Apr-May)	10 (9.9)	48 (10.0)	92 (17.7)
Post Harvest (Jun-Aug)	61 (60.4)	224 (46.6)	173 (33.2)
Short Rain (Sept-Nov)	21 (20.8)	151 (31.4)	173 (33.2)
BMI (kg/m^2^)			
Severe underweight (<16.0)	10 (10.1)	22 (4.6)	41 (7.9)
Underweight (16.0–18.5)	24 (24.2)	95 (19.9)	111 (21.5)
Normal (18.5–25) (ref)	49 (49.5)	286 (59.8)	302 (58.5)
Overweight (>25.0)	16 (16.2)	75 (15.7)	62 (12.0)
WHO HIV Disease Stage			
I or II	24 (25.8)	111 (25.4)	100 (21.1)
III	51 (54.8)	274 (62.7)	306 (64.5)
IV	18 (19.4)	52 (11.9)	68 (14.3)
CD4 T-cell Category (cells/ µL)			
<50	15 (15.0)	76 (16.7)	123 (24.5)
50–100	12 (12.0)	72 (15.8)	113 (22.5)
100–200	49 (49.0)	200 (43.9)	180 (35.7)
>200	24 (24.0)	108 (23.7)	87 (17.3)
Hemoglobin (g/dL)	9.99±2.34	10.25±2.26	10.05±2.23
ART regimen prescribed at baseline*			
d4T, 3TC, NVP	52 (51.5)	278 (57.8)	299 (57.4)
d4T, 3TC, EFV	11 (10.9)	49 (10.2)	58 (11.1)
AZT, 3TC, NVP	6 (5.9)	39 (8.1)	43 (8.3)
AZT, 3TC, EFV	32 (31.7)	115 (23.9)	121 (23.2)
Vitamin D (ng/mL)	16.4±3.15	25.33±2.77	36.97±6.22

d4T = stavudine, AZT = zidovudine, 3TC = lamivudine, NVP = nevirapine, EFV = efavirenz.

* Vitamin D levels obtained before ART initiation.

**Table 2:** Risk factors for low vitamin D (<30 ng/mL).

	Univariate RR (95% CI)	p-value*	Multivariate RR (95% CI)	p-value*
**Sex**				
Female (ref)	1.0		1.0	
Male	0.94 (0.83–1.06)	0.334	1.00 (0.88–1.14)	0.992
**Age (years)**				
Under 30 (ref)	1.0	0.002	1.0	<0.001
30–40	0.85 (0.74–0.98)		0.85 (0.75–0.98)	
40–50	0.68 (0.57–0.80)		0.66 (0.56–0.79)	
Over 50	0.84 (0.68–1.04)		0.79 (0.64–0.98)	
**Season**				
Long Rain (Dec-Mar)	0.72 (0.59–0.87)	<0.001	0.77 (0.63–0.92)	0.005
Harvest (Apr–May)	0.62 (0.50–0.77)	<0.001	0.62 (0.50–0.76)	<0.001
Post Harvest (Jun–Aug) (ref)	1.0		1.0	
Short Rain (Sept–Oct)	0.80 (0.71–0.91)	<0.001	0.80 (0.71–0.91)	0.001
**BMI (kg/m^2^)**				
Severely underweight (<16.0)	0.74 (0.55–0.99)	0.030	0.78 (0.58–1.05)	0.091
Underweight (16.0–18.5)	0.87 (0.73–1.04)		0.90 (0.58–1.08)	
Normal (18.5–25)	0.88 (0.76–1.03)		0.90 (0.77–1.04)	
Overweight (> = 25.0) (ref)	1.0		1.0	
**WHO HIV Disease Stage**				
I or II	1.13 (0.93–1.38)	0.132	1.04 (0.85–1.28)	0.564
III	1.02 (0.84–1.22)		0.98 (0.82–1.17)	
IV (ref)	1.0		1.0	
**CD4 T-cell count (cells/** **µL)**				
<50	0.71 (0.58–0.85)	<0.001	0.69 (0.57–0.83)	<0.001
50–100	0.71 (0.69–0.86)		0.70 (0.58–0.85)	
100–200	0.96 (0.84–1.10)		0.92 (0.71–1.20)	
>200 (ref)	1.0		1.0	
**Hemoglobin (g/dL)**				
<8.5	0.92 (0.79–1.09)	0.368	0.95 (0.73–1.22)	0.512
8.5–10.9	0.96 (0.84–1.09)		1.02 (0.87–1.21)	
< = 11 (ref)	1.0		1.0	

* p-value for test for trend for rank variables.

**Figure 1:**
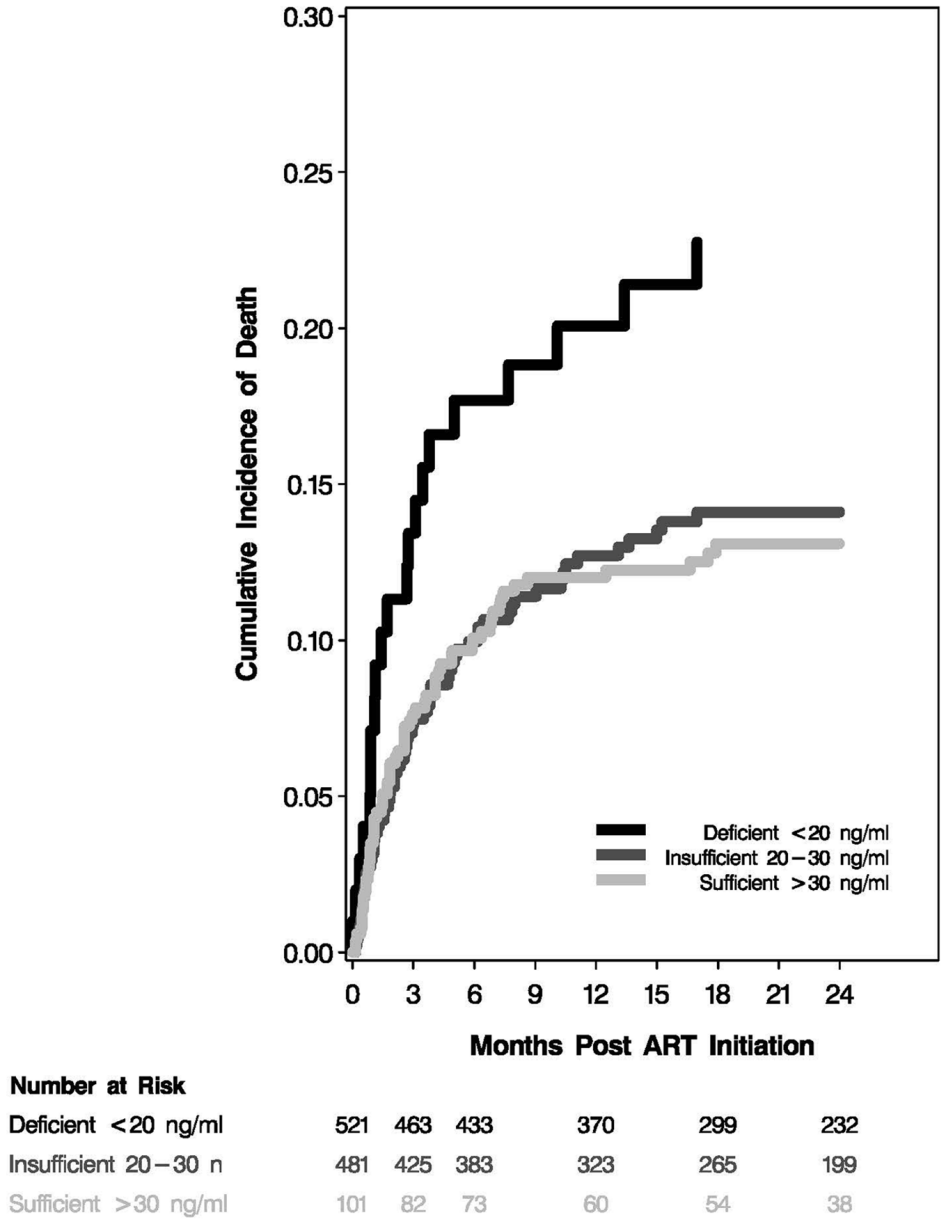
Kaplan-Meier estimation of progression to all-cause mortality by vitamin D status.

The median follow-up time was 20.6 months (IQR: 8.4–33.8), during which 151 deaths were recorded. A crude Kaplan-Meir curve for all-cause mortality by vitamin D status is presented in Figure 1 showing that at 24 months post ART initiation 22.8% of the individuals with deficient vitamin D levels died as compared to 14.1% and 13.1% of individuals with insufficient and deficient levels of vitamin D, respectively.

**Figure 2:**
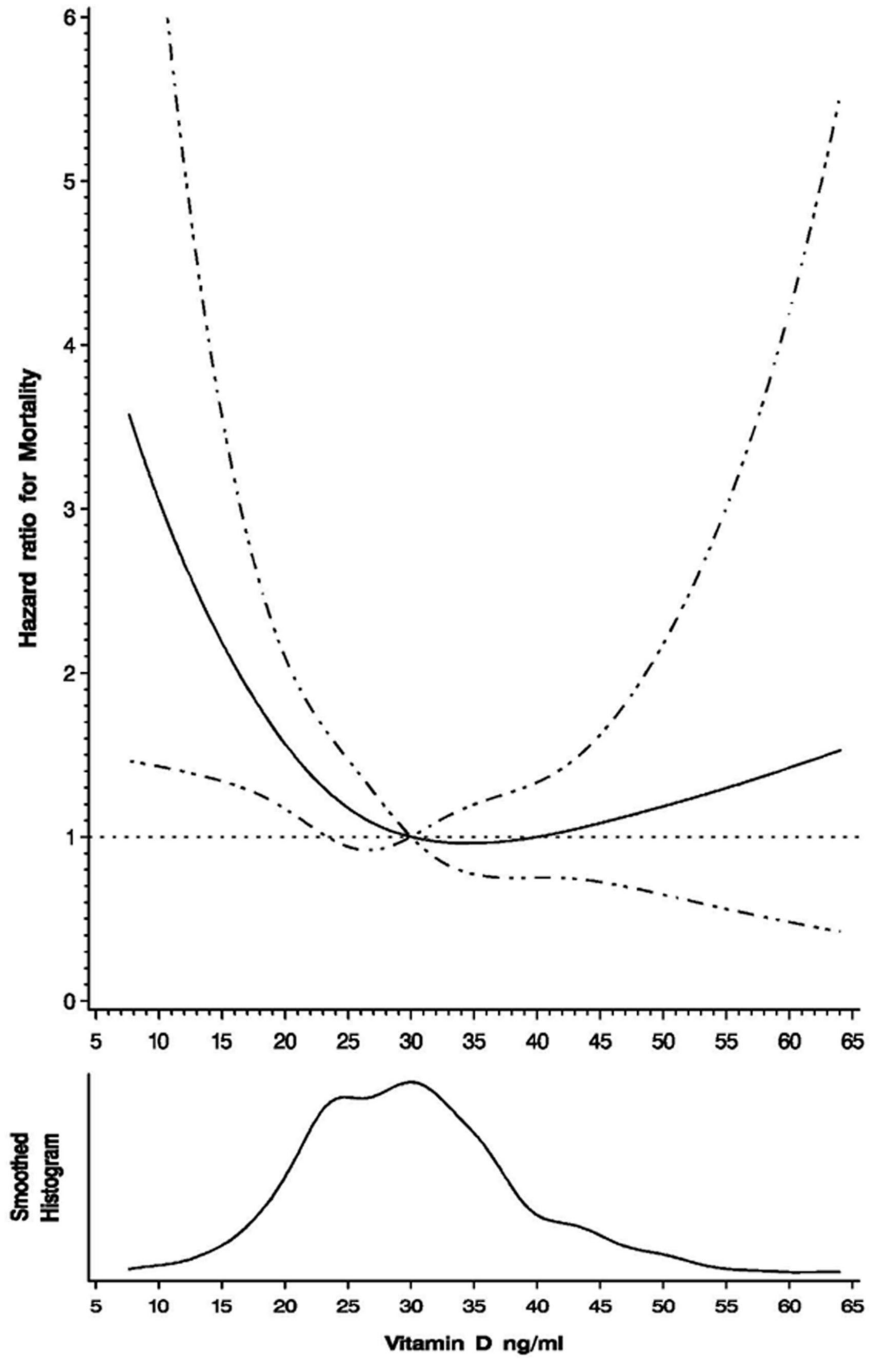
Restricted cubic spline analysis illustrating the shape of the serum 25(OH)D and all-cause mortality association (hazard ratio) continuously with 30 ng/mL as the referent level. Adjusted for baseline sex, age, season, BMI, WHO HIV disease stage, and CD4 T-cell count.

The results of univariate and multivariate proportional hazard analyses for mortality outcomes by vitamin D status are presented in Table 3. After multivariate adjustment, the hazard of death was 2.00 (95% CI: 1.19–3.37; p = 0.009) times greater for individuals with vitamin D deficiency at baseline compared to vitamin D sufficient individuals. There was no significant increase for individuals with insufficient levels of vitamin D compared to sufficient individuals (HR: 1.24; 95%: 0.87–1.78; p = 0.240). Further, when analyzing 25(OH)D as a continuous variable we found a non-linear relationship with increasing risk of all-cause mortality below 25 ng/mL and seemingly no increased benefit to baseline vitamin D levels greater than 30 ng/mL (Figure 2). No significant effect modification by baseline CD4 T-cell count, age, sex, randomized multivitamin regimen, ART regimen or specific antiretroviral drug was found. There was also no indication that the hazard of death for vitamin D deficiency (p = 0.97) or insufficiency (p = 0.40) changed over time.

**Table 3:** Hazard ratio for mortality by vitamin D status.

	Vitamin D Deficient (<20 ng/mL)	p-value	Vitamin D Insufficient(20–30 ng/mL)	p-value	Vitamin D Sufficient (>30 ng/mL)
Unadjusted All-Cause Mortality (n = 151)	1.83 (1.12–3.30)	0.016	1.09 (0.77–1.53)	0.633	1.0 (ref)
Adjusted* All-Cause Mortality(n = 151)	2.00 (1.19–3.37)	0.009	1.24 (0.87–1.78)	0.240	1.0 (ref)

* Adjusted for baseline sex, age, season, BMI, WHO HIV disease stage, and CD4 T-cell count.

**Figure 3:**
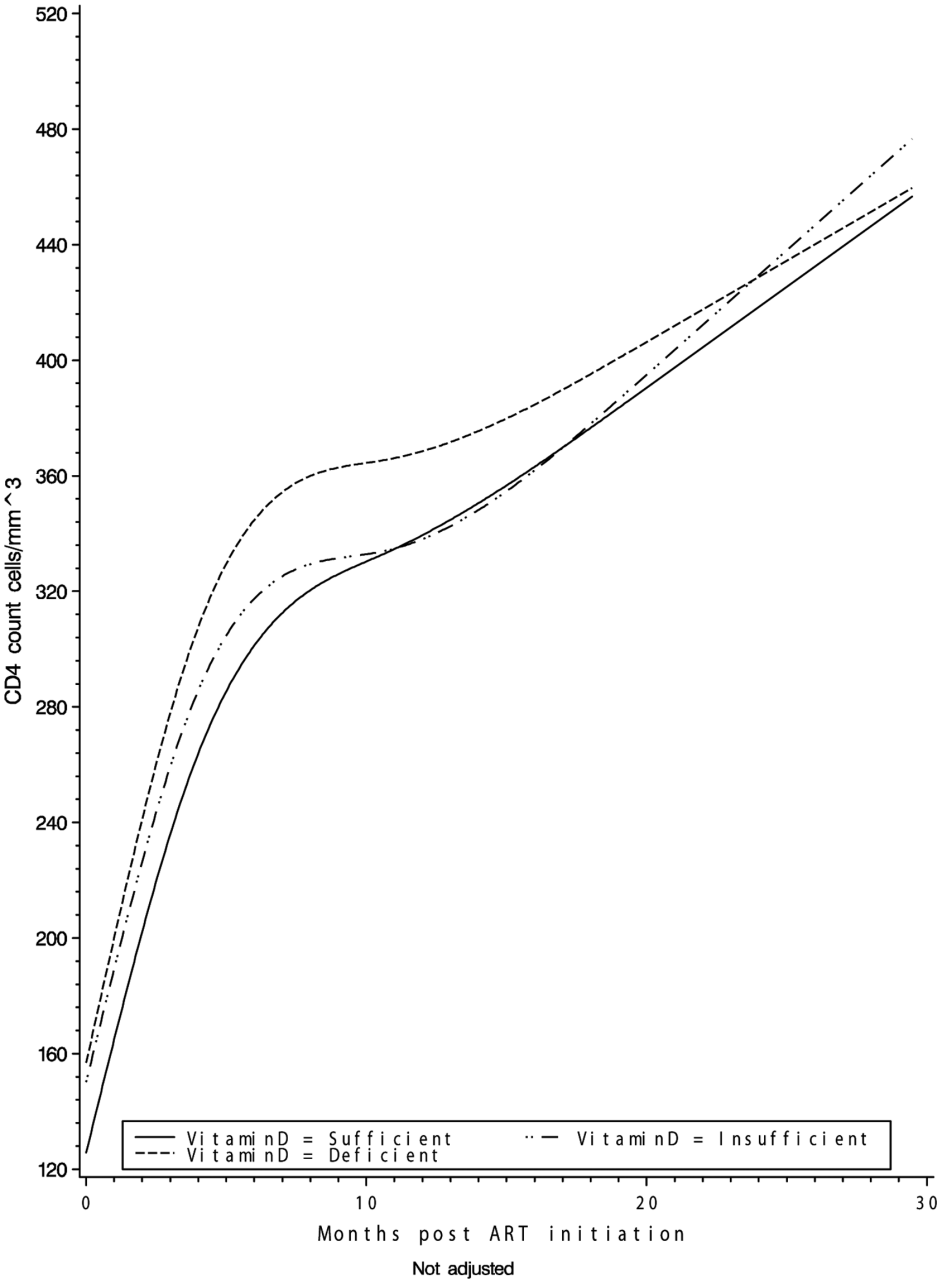
Crude CD4 T-cell count overtime by vitamin D status.

A total of 875 individuals had 2 or more CD4 T-cell count measurements during the study period. As expected the change in CD4 T-cell count post ART initiation was non-linear. Participants, regardless of vitamin D status, experienced dramatic increases in CD4 T-cell counts during the first 6 months of ART with more modest gains thereafter (Figure 3). In crude models adjusting solely for time between visits, the trajectory of change in CD4 T-cell count between consecutive visits did not differ in vitamin D deficient (p = 0.403) or insufficient (p = 0.885) individuals as compared to those with sufficient vitamin D levels at baseline. There were no differences in CD4 T-cell trajectory in multivariate models further adjusting for sex, age, WHO HIV disease stage, BMI, and ART regimen for deficient (p = 0.374) or insufficient individuals (p = 0.943) as compared to sufficient individuals. No significant effect modification by age, sex, randomized multivitamin regimen, ART regimen or specific antiretroviral drug was found.

## Discussion

In this study we found individuals with deficient levels of vitamin D (<20 ng/mL) at ART initiation had significantly increased risk of mortality as compared to individuals with sufficient levels of vitamin D (>30 ng/mL), whereas there was no significant increase for individuals with vitamin D insufficiency (20–30 ng/mL). The benefit of sufficient vitamin D does not appear to be mediated through improved immune reconstitution as there was no association between vitamin D status and change in CD4 T-cell count after ART initiation. We also found no effect modification by any factor, including ART regimen, in either mortality or CD4 T-cell analyses.

The mortality results of this study are consistent with previous cohort studies. In the EUROSIDA study adults in the lowest tertile of 25(OH)D (<12 ng/mL) had significantly increased incidence of mortality as compared adults in the middle (12.1–20 ng/mL) and highest tertile (≥20 ng/mL), but there was no significant difference between the middle and highest tertiles [21]. Individuals in the EUROSIDA cohort had markedly low levels of vitamin D with 89% of individuals below 30 ng/mL and as a result, the study likely had inadequate power to examine the relationship using clinical classifications with sufficiency (>30 ng/mL) defining the referent group. The second cohort study consisting of Tanzanian pregnant women did not find a significant association of low vitamin D with all-cause mortality using a cut-off of 32 ng/mL, but found a significant difference when comparing women in the lowest quintile of vitamin D to the highest quintile [16]. As opposed to the EUROSIDA study, these Tanzanian pregnant women may have had too high levels of vitamin D to find a significant difference with these clinical cutoffs since only 40% of women had levels below 32 ng/mL with very few below 20 ng/mL. Our categorical analyses in this study found individuals with vitamin D deficiency (<20 ng/mL) had significantly increased risk of mortality as compared to those with sufficient vitamin D levels (≥30 ng/mL), while individuals with vitamin D insufficiency (20–30 ng/mL) appeared to have more moderately elevated mortality risk, but results were not statistically significant. Nevertheless, our analysis of 25(OH)D as a continuous exposure suggests mortality risk begins to increase at levels below 25 ng/mL. Accordingly, using the standard vitamin D status classifications (<20, 20–30, ≥30 ng/mL) may make cross study comparison less complicated; however, more research with synthesis of results in different settings is needed to determine if these standard cut-offs based on calcium homeostasis and parathyroid hormone (PTH) regulation are directly applicable to the vitamin D and HIV mortality relationship, especially for individuals in the 20–30 ng/mL 25(OH)D range [29], [31]–[33].

The beneficial effect of vitamin D on mortality for HIV-infected individuals may be partially explained by its regulatory effect on the innate immune system [37], [38]. Recent studies have found vitamin D is required for proper functioning of an interferon-gamma mediated pathway in macrophages which leads to autophagy, phagosomal maturation, and antimicrobial activity [5], [6]. Expression of antimicrobial peptides, including cathelicidin and defensin β2, are also up-regulated in macrophages stimulated by 1,25(OH)_2_D, however if 1,25(OH)_2_D levels fall below 20 ng/mL the cathelicidin response is not initiated [38]–[40]. Consequently, individuals with vitamin D deficiency may have impaired innate immune responses which result in increased incidence or more severe opportunistic infections.

As for adaptive immune responses, 1,25(OH)_2_D has been shown to reduce T-cell proliferation due to suppression of interleukin (IL)-2 and direct inhibition of NFATp/AP-1 formation *in vitro* [41], [42]. Consistent with these findings we found that sufficient levels of vitamin D were associated with lower CD4 T-cell counts at baseline, but there was no difference in CD4 T-cell count change over time after ART initiation by vitamin D status. In contrast, previous cross-sectional studies have found low vitamin D levels were associated with low CD4 T-cell counts, which is also biologically plausible since individuals with low CD4 T-cell counts have decreased exposure to sunlight or increased risk of opportunistic infections that may lead to increased immune activation and utilization of vitamin D by immune cells [43]–[46]. A possible explanation for these seemingly conflicting results is that the 25(OH)D and CD4 T-cell relationship takes on a U-shaped curve. Cross-sectional studies that have found an association of low vitamin D and low CD4 T-cell count have mainly been conducted in high latitude settings where very few individuals have 25(OH)D levels above 30 ng/mL [43]–[45]. As a result, the reference group in these studies may consist of individuals with too low of 25(OH)D levels to experience a pronounced reduction in CD4 T-cell proliferation, whereas in our study about 50% of individuals have 25(OH)D levels above 30 ng/mL the reference group may have 25(OH)D levels where CD4 T-cell proliferation is significantly inhibited.

In longitudinal analyses we found no association between vitamin D and change in CD4 T-cell counts after ART initiation. These results are consistent with a previous observational cohort study of German adults primarily receiving ART which found no difference in median CD4 T-cell recovery rates during follow-up for the total cohort or when stratified by baseline CD4 T-cell count [47]. In contrast, a cohort study of US adults receiving ART found low vitamin D was significantly associated with decreased change in CD4 T-cell count (current minus nadir) overtime [48]. Nevertheless, the authors adjusted for nadir CD4 T-cell count in analyses, which can produce bias if vitamin D is associated with baseline health status and also vitamin D was measured at least 24 weeks after start of ART which increases the risk of reverse causation [49]. Further, two small randomized controlled trials in HIV-infected children on ART also reported no effect of vitamin D supplementation on change in CD4 T-cell count overtime [50], [51]. Overall, our risk factor analysis and other cross-sectional studies have produced varying results for the impact of vitamin D on CD4 T-cell count, but our prospective analysis along with other longitudinal studies and randomized trials suggest there is no pronounced difference in change in CD4 T-cell counts during the initial months of ART by vitamin D status.

There may also be immunomodulatory benefits to maintaining sufficient vitamin D for other aspects of adaptive immune responses. In addition to IL-2 suppression, vitamin D also reduces production of other proinflammatory Th1 and Th17 cytokines, while also enhancing production of Th2 promoting cytokines including: IL-4, IL-5, and IL-10 [52]–[55]. 1,25(OH)_2_D has also been shown to reduce B-cell proliferation, plasma differentiation, and immunoglobulin production *in vitro* [56], [57]. Further, 1, 25(OH)_2_D can also suppress the response of Th17 cells, which cause inflammation and tissue destruction and also induce Foxp3+ T regulatory cells, which suppress excessively strong CD4 T-cell proliferation [58], [59]. The sum of these actions will likely decrease inflammation, tissue damage, and reduce excessively strong immune responses to opportunistic infections in individuals with adequate levels of vitamin D which may in turn lead to reduced mortality [60], [61]. Overall, there are multiple mechanisms by which vitamin D may support innate or adaptive immune responses to opportunistic infections and reduce subsequent mortality.

Antiretroviral drugs can alter vitamin D metabolism and result in decreased circulating 25(OH)D [19]. The non-nucleoside reverse transcriptase inhibitor (NNRTI) efavirenz induces 24-hydroxylase, which hydrolyzes active vitamin D to its inactive form [62]. A prospective study of individuals initiating ART in the US found individuals receiving regimens containing efavirenz had 1.8 times the risk of developing 25(OH)D levels ≤15 ng/mL as compared to individuals initiating regimens containing protease inhibitors [63]. Further, the Monet trial found that patients who switched from regimens containing efavirenz and/or zidovudine to darunavir/ritonavir experienced increases in serum 25(OH)D [64]. Protease inhibitors have also been noted to alter vitamin D metabolism, but we were unable to investigate this class of antiretroviral drugs as they were not used in Tanzania during the study period [65], [66]. We did not find effect modification of mortality or CD4 T-cell associations by efavirenz-containing or other ART regimens in this analysis. However, we may have had limited statistical power to detect multiplicative interaction, and it is plausible that vitamin D insufficient individuals who decrease serum 25(OH)D to deficient levels as a result of ART or use of particular antiretroviral drugs may experience slightly increased mortality relative to those on ART therapy who maintain sufficient serum 25(OH)D levels.

A major strength of this study is the use of clinically defined vitamin D cut-offs, which can make direct comparison of studies easier. We also quantified 25(OH)D with HPLC-MS/MS, a reference method, which will reduce non-differential misclassification of vitamin D status or even bias if individuals with more severe HIV disease produce metabolites or other molecules which may interfere with 25(OH)D measurement using radioimmunoassy (RIA) [15], [67] Furthermore, measuring 25(OH)D at ART initiation allows for examination of effect modification by ART regimen. It is unlikely interaction would be detected in individuals already receiving ARTs at the time of vitamin D measurement, since individuals would have already experienced a reduction in circulating 25(OH)D as a result of treatment.

The study also has several important limitations. First, 25(OH)D was measured at a single time point and we are unable to determine whether deficient vitamin D levels at a single time point or long term deficiency is biologically relevant. Nevertheless, we found no change in the association of vitamin D deficiency at baseline and mortality over time, which suggests a single vitamin D measurement at ART initiation may be able to indicate which patients may benefit from vitamin D supplementation. Second, the results of this study may not be directly generalizable to other HIV-infected populations, including pregnant women or children on ART. A previous study in HIV-infected pregnant women not receiving ART in Tanzania found having vitamin D levels above 32 ng/mL provided additional benefit; however, this study indicated no benefit to having levels above 30 ng/mL for men and non-pregnant women on ART in the same setting [17]. Further, we presented an observational study from which reverse causation cannot be ruled out as advanced HIV disease or other co-morbidities may have led to lower vitamin D levels. We controlled multivariate analyses for multiple indicators of disease severity (CD4 T-cell count, WHO HIV disease stage, BMI) and if residual confounding by these factors is present we may actually underestimate the association of vitamin D and mortality. Nevertheless, unmeasured confounding by other factors like tuberculosis or hepatitis C infection at baseline may still produce biased results [68], [69].

This study suggests that deficient vitamin D levels may lead to increased mortality in HIV-infected men and non-pregnant women receiving ART and this association does not appear to be mediated by impaired CD4 T-cell reconstitution. However, due to the observational design of this study a causal relationship cannot be concluded. Accordingly, randomized controlled trials are warranted to determine the safety and efficacy of vitamin D supplementation for HIV-infected individuals receiving ART.
